# BAP1 suppresses lung cancer progression and is inhibited by miR-31

**DOI:** 10.18632/oncotarget.7328

**Published:** 2016-02-11

**Authors:** Mengchao Yu, Hongwei Liang, Zheng Fu, Xueliang Wang, Zhicong Liao, Yong Zhou, Yanqing Liu, Yanbo Wang, Yeting Hong, Xinyan Zhou, Xin Yan, Min Yu, Miao Ma, Weijie Zhang, Baoliang Guo, Jianguo Zhang, Ke Zen, Chen-Yu Zhang, Tao Wang, Qipeng Zhang, Xi Chen

**Affiliations:** ^1^ State Key Laboratory of Pharmaceutical Biotechnology, Collaborative Innovation Center of Chemistry for Life Sciences, Jiangsu Engineering Research Center for MicroRNA Biology and Biotechnology, NJU Advanced Institute for Life Sciences (NAILS), School of Life Sciences, Nanjing University, Nanjing, Jiangsu, China; ^2^ Department of Cardio-Thoracic Surgery, Nanjing Drum Tower Hospital Affiliated to Medical School of Nanjing University and Nanjing Multi-Center Biobank, Nanjing, Jiangsu, China; ^3^ Department of Respiratory Medicine, Nanjing Drum Tower Hospital Affiliated to Medical School of Nanjing University, Nanjing, Jiangsu, China; ^4^ Department of General Surgery, Nanjing Drum Tower Hospital Affiliated to Medical School of Nanjing University, Nanjing, Jiangsu, China; ^5^ Department of General Surgery, The Second Affiliated Hospital of Harbin Medical University, Harbin, Heilongjiang, China

**Keywords:** lung cancer, BAP1, miR-31, proliferation, apoptosis

## Abstract

BRCA1-associated protein-1 (BAP1) is an important nuclear-localized deubiquitinating enzyme that serves as a tumor suppressor in lung cancer; however, its function and its regulation are largely unknown. In this study, we found that BAP1 protein levels were dramatically diminished in lung cancer tissues while its mRNA levels did not differ significantly, suggesting that a post-transcriptional mechanism was involved in BAP1 regulation. Because microRNAs (miRNAs) are powerful post-transcriptional regulators of gene expression, we used bioinformatic analyses to search for miRNAs that could potentially bind BAP1. We predicted and experimentally validated miR-31 as a direct regulator of BAP1. Moreover, we showed that miR-31 promoted proliferation and suppressed apoptosis in lung cancer cells and accelerated the development of tumor growth in xenograft mice by inhibiting BAP1. Taken together, this study highlights an important role for miR-31 in the suppression of BAP1 in lung cancer cells and may provide insights into the molecular mechanisms of lung carcinogenesis.

## INTRODUCTION

Lung cancer is the leading cause of death from malignant disease in the developed world for men and women, and non-small cell lung cancer (NSCLC) accounts for approximately 85% of all lung cancer subtypes [1]. Because lung cancer does not usually become clinically evident until it reaches an advanced stage, most patients are diagnosed with metastatic and advanced disease, and only a small proportion is eligible for surgical resection and radical treatment [2]. Thus, new treatment strategies are needed. Recently, the landscape of lung cancer therapy has been significantly altered by the development of targeted therapy. Although some drugs targeting epidermal growth factor receptor (EGFR) mutations have been developed, most advanced NSCLC is still incurable, and new targets for anticancer drugs are in demand.

BRCA1-associated protein 1 (BAP1) was originally identified as a protein that interacted with the RING finger domain of the breast cancer susceptibility gene product BRCA1 [3]. BAP1 is a deubiquitinating enzyme with a ubiquitin carboxy-terminal hydrolase (UCH) domain that gives BAP1 its deubiquitinase activity. BAP1 has been reported to participate numerous cellular processes, such as cell fate determination, stem cell pluripotency and other developmental processes. For example, BAP1 can enhance progression through the G1-S checkpoint and subsequently induce cell death by a process with similarities to both apoptosis and necrosis [4]. Additionally, BAP1 inhibits cell proliferation by deubiquitinating host cell factor-1 (HCF1) [5]. In cancer, BAP1 can function as both a tumor suppressor and a metastasis suppressor. Overexpression of BAP1 was shown to suppress tumor cell expansion in mouse xenografts [4]. In patients with advanced NSCLC, high BAP1 expression was found to be associated with a lack of lymph node metastasis and a longer median survival time [6], and deletions in the BAP1 gene were present in lung adenocarcinoma and other types of human cancers [7]. However, the roles of BAP1 in the initiation and progression of human cancers remain poorly understood. Furthermore, 25% of tumors without identified BAP1 mutations did not display any immunohistochemical staining for BAP1 [8], suggesting the possibility of another subset of tumors with a functional BAP1 loss that arose by other mechanisms.

MicroRNAs (miRNA) are recently discovered small (19∼22 nucleotide) noncoding RNAs that function as the repressors of gene activity in animals and plants [9, 10]. Functionally, miRNAs bind to complementary sequences in the 3′ untranslated region (3′-UTR) of target gene transcripts, leading to mRNA degradation and/or translational repression; as a result, the expression and function of the target gene is suppressed [11]. Aberrant expression of miRNAs has been implicated in several human cancers, and each cancer type has a unique miRNA expression pattern [12]. The specific expression patterns of miRNAs in cancers enable miRNAs to serve as biomarkers for cancer risk, diagnosis and prognosis, and even as miRNA-based therapeutic targets. Importantly, miRNAs can suppress multiple tumor suppressor genes or oncogenes during carcinogenesis, thereby functioning as oncogenes or tumor suppressors, respectively. Among the miRNAs correlated with carcinogenesis, miR-31 is one of the most important. miR-31 was located on chromosome 9p21.3 and was first identified in HeLa cells [13]. Comprehensive miRNA microarray analyses on pulmonary adenocarcinomas revealed that miR-31 was one of the most overexpressed miRNAs, and knockdown of miR-31 significantly reduced lung cancer cell growth [14]. Moreover, miR-31 was found to be increased in lung adenocarcinomas from patients with lymph node metastases compared to those without lymph node metastases [15]. These studies suggest a possible oncogenic role for miR-31 in lung tumorigenesis, although its detailed mechanism of action remains to be elucidated.

In this study, we identified BAP1 as a direct target gene of miR-31 and showed that miR-31 inhibited BAP1 expression. Consequently, miR-31 promoted the proliferation and suppressed the apoptosis of lung cancer cells *in vitro* and accelerated lung tumor growth *in vivo*.

## RESULTS

### Downregulation of BAP1 protein but not mRNA expression in human lung cancer tissues

**Figure 1:**
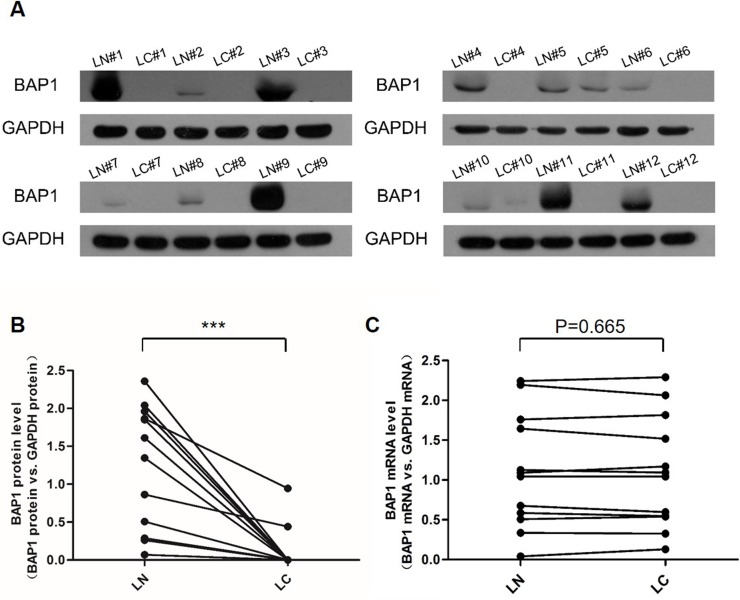
Expression patterns of BAP1 in human lung cancer tissues **A.** and **B.** Western blotting analysis of the expression levels of the BAP1 protein in 12 pairs of lung cancer tissue (LC) and lung noncancerous tissue (LN) samples. **A.** representative image; **B.** quantitative analysis (BAP1 protein *vs*. GAPDH protein). **C.** Quantitative RT-PCR analysis of the expression levels (BAP1 mRNA *vs*. GAPDH mRNA) of BAP1 mRNA in the same 12 pairs of LC and LN samples. (*** *p* < 0.005).

We first determined the expression patterns of BAP1 in human lung cancer tissues. After measuring the BAP1 protein levels in 12 pairs of lung cancer tissues and adjacent noncancerous tissues (The clinical features of the patients are listed in Table 1), we showed that the BAP1 protein levels were dramatically diminished in the lung cancer tissues (Figure 1A and 1B). In contrast, the BAP1 mRNA levels did not differ significantly between the cancer and noncancerous tissues (Figure 1C). This disparity between BAP1 protein and mRNA expression in lung cancer tissues strongly suggests that a post-transcriptional mechanism is involved in BAP1 regulation.

**Table 1:** Clinical features of lung cancer patients

	Age	Gender	Tumor subtype	Pathological Stage
Case #1	60	Female	Adenocarcinoma	II A
Case #2	58	Male	Adenocarcinoma	I B
Case #3	67	Female	Adenocarcinoma	III A
Case #4	60	Male	Squamous cell carcinoma	I B
Case #5	70	Male	Adenocarcinoma	I B
Case #6	70	Male	Adenocarcinoma	I
Case #7	59	Male	Adenocarcinoma	III A
Case #8	59	Male	Adenocarcinoma	
Case #9	65	Female	Adenocarcinoma	III A
Case #10	76	Male	Adenocarcinoma	
Case #11	72	Male	Squamous cell carcinoma	III A
Case #12	76	Male	Adenocarcinoma	

### Prediction of BAP1 as a target gene of miR-31

**Figure 2:**
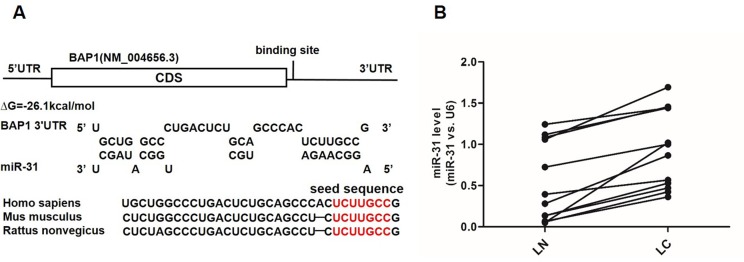
Prediction of the miR-31 binding site within the BAP1 3′-UTR **A.** Schematic description of the hypothetical duplexes formed by the interactions between the binding site in the BAP1 3′-UTR (top) and miR-31 (bottom). The seed regions of miR-31 and the seed-recognition sites in the BAP1 3′-UTR are indicated in red. All nucleotides in the seed-recognition sites are completely conserved in several species. The predicted free energy values of each hybrid are indicated. **B.** Quantitative RT-PCR analysis of the expression levels (miR-31 *vs*. U6) of miR-31 in the same 12 pairs of LC and LN samples. (*** *p* < 0.005).

Because miRNAs play important roles in post-transcriptional regulation, it is quite likely that miRNAs inhibit BAP1 expression in human lung cancer. Next, three computational algorithms (TargetScan [16], miRanda [17] and PicTar [18]) were used in combination to identify potential miRNAs that bound BAP1. Among the candidate miRNAs, miR-31 was predicted to be a BAP1 regulator by all three algorithms and was selected for experimental verification. The predicted conjugation between miR-31 and the binding site within the BAP1 3′-UTR is illustrated in Figure 2A. As shown in this figure, the 3′-UTR of BAP1 contained one conserved binding site for miR-31. The minimum free energy value of the hybrid was −26.5 kcal/mol, which was well within the range of genuine miRNA-target pairs. Moreover, there was perfect base-pairing between the seed region (the core sequence that encompasses the first 2-8 bases of the mature miRNA) and the cognate target.

### Detection of an inverse correlation between miR-31 and BAP1 levels in lung cancer tissues

Because miRNAs are generally thought to have expression patterns that are opposite to that of their targets [9, 19, 20], we investigated whether miR-31 expression was inversely correlated with BAP1 expression in lung cancer. We measured the expression levels of miR-31 in the same 12 pairs of lung cancer tissues and corresponding noncancerous tissues and found that the miR-31 levels were consistently higher in the cancer tissues (Figure 2B). The results strongly indicated that a typical miR-31-mediated post-transcriptional regulation mechanism was involved in BAP1 repression.

### Validation of BAP1 as a direct target of miR-31

**Figure 3:**
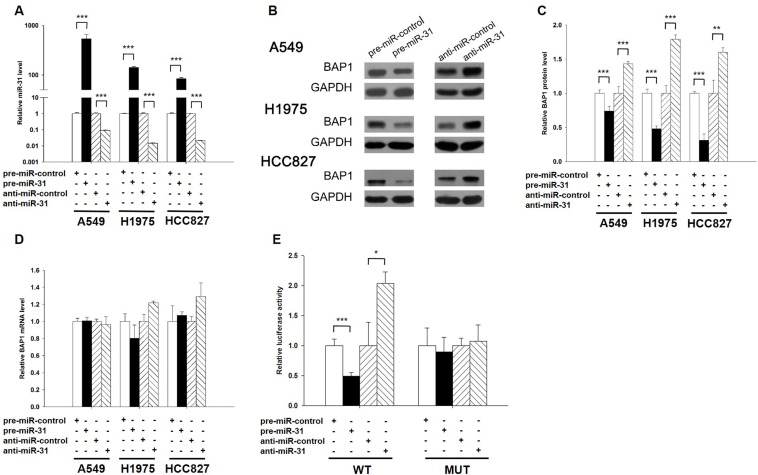
BAP1 is a direct target of miR-31 **A.** Quantitative RT-PCR analysis of the expression levels of miR-31 in A549, H1975 and HCC827 cells transfected with equal doses of the miR-31 mimic (pre-miR-31), miR-31 inhibitor (anti-miR-31) or scrambled negative control RNA (pre-miR-control or anti-miR-control). **B.** and **C.** Western blotting analysis to detect BAP1 protein levels in A549, H1975 and HCC827 cells transfected with equal doses of the miR-31 mimic, miR-31 inhibitor or scrambled negative control RNA. B: representative image; **C.** quantitative analysis. **D.** Quantitative RT-PCR analysis of BAP1 mRNA levels in A549, H1975 and HCC827 cells transfected with equal doses of the miR-31 mimic, miR-31 inhibitor or scrambled negative control RNA. **E.** Direct recognition of the BAP1 3′-UTR by miR-31. Firefly luciferase reporters containing either wild-type (WT) or mutant (MUT) miR-31 binding sites in the BAP1 3′-UTR were co-transfected into A549 cells with equal doses of the miR-31 mimic, miR-31 inhibitor or scrambled negative control RNA. Twenty-four hours post-transfection, the cells were assayed using a luciferase assay kit. Firefly luciferase values were normalized to β-galactosidase activity, and the results were calculated as the ratio of firefly luciferase activity in the miR-31-transfected cells normalized to the negative control RNA-transfected cells. The results are presented as the mean ± S.D. of three independent experiments. (* *p* < 0.05; ** *p* < 0.01; *** *p* < 0.005).

The correlation between miR-31 and BAP1 was examined by evaluating BAP1 expression in human lung adenocarcinoma A549 cells after overexpression or knockdown of miR-31. In these experiments, miR-31 overexpression was achieved by transfecting A549 cells with a miR-31 mimic (synthetic double-stranded RNA oligonucleotide mimicking precursor of miR-31), whereas miR-31 knockdown was achieved by transfecting A549 cells with a miR-31 inhibitor (chemically modified antisense oligonucleotide designed to sequester mature miR-31). The efficient overexpression or knockdown of miR-31 in A549 cells is shown in Figure 3A. As anticipated, the expression of the BAP1 protein was significantly reduced by the introduction of miR-31, whereas the miR-31 inhibitor significantly increased the BAP1 protein levels in A549 cells (Figure 3B and 3C). To determine the extent to which miR-31 influenced BAP1 expression, we repeated the above experiments and examined the expression of the BAP1 mRNA after transfection. Overexpression or knockdown of miR-31 did not decrease BAP1 mRNA levels (Figure 3D). To demonstrate the robustness of the test, we repeated the above experiments in additional lung cancer cell lines (H1975 and HCC827) and observed consistent results (Figure 3A-3D). To determine whether the negative regulatory effects that miR-31 exerted on BAP1 expression were mediated through the binding of miR-31 to the presumed site in the BAP1 3′-UTR, we fused the region of the BAP1 3′-UTR that contained the presumed miR-31 binding site downstream of the firefly luciferase reporter plasmid. The resulting plasmid was transfected into A549 cells along with the miR-31 mimic, miR-31 inhibitor or scrambled negative control RNA. As expected, overexpression of miR-31 resulted in an approximately 50% reduction in luciferase reporter activity compared with cells treated with the control mimic, whereas inhibition of miR-31 resulted in a two-fold increase in reporter activity compared with cells transfected with the control inhibitor (Figure 3E). Next, we constructed a mutant plasmid by introducing point mutations into the corresponding complementary seed site in the BAP1 3′-UTR to eliminate the predicted miR-31 binding site. This mutated luciferase reporter was unchanged by either the overexpression or knockdown of miR-31 (Figure 3E). This finding suggests that the binding site strongly contributes to the miRNA-mRNA conjugation and participates the post-transcriptional repression of BAP1 expression. In conclusion, our results demonstrate that miR-31 directly recognizes and binds to the 3′-UTR of the BAP1 transcript and inhibits BAP1 translation.

### miR-31 promotes proliferation and suppresses apoptosis in lung cancer cells by inhibiting BAP1

**Figure 4:**
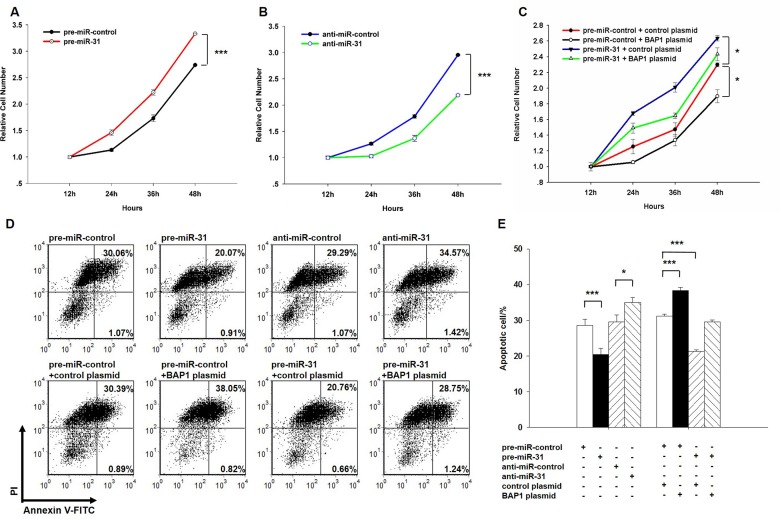
Effect of miR-31 and BAP1 on the proliferation and apoptosis of lung cancer cells **A.** A cell proliferation assay was performed 12, 24, 36 and 48 hours after the transfection of A549 cells with equal doses of the miR-31 mimic or scrambled negative control RNA. **B.** The cell proliferation assay was performed 12, 24, 36 and 48 hours after the transfection of A549 cells with equal doses of the miR-31 inhibitor or scrambled negative control RNA. **C.** The cell proliferation assay was performed 12, 24, 36 and 48 hours after the transfection of A549 cells with equal doses of the pre-miR-control plus control plasmid, pre-miR-control plus BAP1 overexpression plasmid, miR-31 mimic plus control plasmid, or miR-31 mimic plus BAP1 overexpression plasmid. **D.** and **E.** The apoptosis assay was performed 24 hours after the transfection of A549 cells with equal doses of the miR-31 mimic, miR-31 inhibitor or scrambled negative control RNA or with equal doses of the pre-miR-control plus control plasmid, pre-miR-control plus BAP1 overexpression plasmid, miR-31 mimic plus control plasmid, or miR-31 mimic plus BAP1 overexpression plasmid. **D.** representative image; **E.** quantitative analysis. The results of cell proliferation are presented as the mean ± S.E. of three independent experience, and other results are presented as the mean ± S.D. of three independent experiments. (* *p* < 0.05; *** *p* < 0.005).

Next, we analyzed the biological consequences of the miR-31-driven repression of BAP1 expression in lung cancer cells. We first evaluated the effects of miR-31 on the proliferation and apoptosis of A549 cells using the cell counting Kit-8 assay and apoptosis assay. In support of the hypothesis that miR-31 functions as an oncogenic miRNA, A549 cells transfected with the miR-31 mimic showed increased proliferation (Figure 4A); in contrast, knockdown of miR-31 had the opposite effect on cell proliferation (Figure 4B). The apoptosis assay showed that the percentage of apoptotic cells was significantly lower in cells transfected with miR-31 and higher in cells transfected with the miR-31 inhibitor (Figure 4D and 4E). These results suggest that miR-31 may synergistically promote proliferation and suppress apoptosis in lung cancer cells. Next, we investigated whether the overexpression or knockdown of BAP1 would have an impact on cell proliferation and apoptosis in A549 cells. To knock down BAP1, three siRNA sequences binding different sites of the BAP1 cDNA were designed, and the sequence with the best interfering effect (si-BAP1#3) was selected and transfected into A549 cells. To overexpress BAP1, an expression plasmid designed to specifically express the full-length BAP1 ORF without the miR-31-responsive 3′-UTR was constructed and transfected into A549 cells. The efficient overexpression or knockdown of BAP1 in A549 cells is shown in Supplementary Figure 1. As anticipated, A549 cells transfected with the BAP1 siRNA proliferated at a higher rate (Supplementary Figure 2A), whereas BAP1 overexpression decreased cell proliferation (Supplementary Figure 2B). Likewise, A549 cells transfected with the BAP1 siRNA showed suppression of cell apoptosis (Supplementary Figure 2C and 2D), whereas overexpression of BAP1 significantly promoted cell apoptosis (Supplementary Figure 2E and 2F). Thus, miR-31 and BAP1 had opposing effects on cell proliferation and apoptosis. Finally, the cells transfected with miR-31 and the BAP1 overexpression plasmid exhibited significantly lower proliferation rates compared with the cells transfected with miR-31 and the control plasmid (Figure 4C), suggesting that miR-31-resistant BAP1 could rescue the suppression of BAP1 by miR-31 and attenuate the proliferative effect of miR-31. Similarly, when A549 cells were simultaneously transfected with miR-31 and the BAP1 overexpression plasmid, BAP1 dramatically rescued the suppressive effect of miR-31 on cell apoptosis (Figure 4D and 4E). These results demonstrate that BAP1 is crucial for the proliferation and apoptosis of lung cancer cells and that miR-31 can promote cell proliferation and inhibit cell apoptosis by silencing BAP1.

### The influence of miR-31 and BAP1 on the growth of lung cancer cells *in vivo*

**Figure 5:**
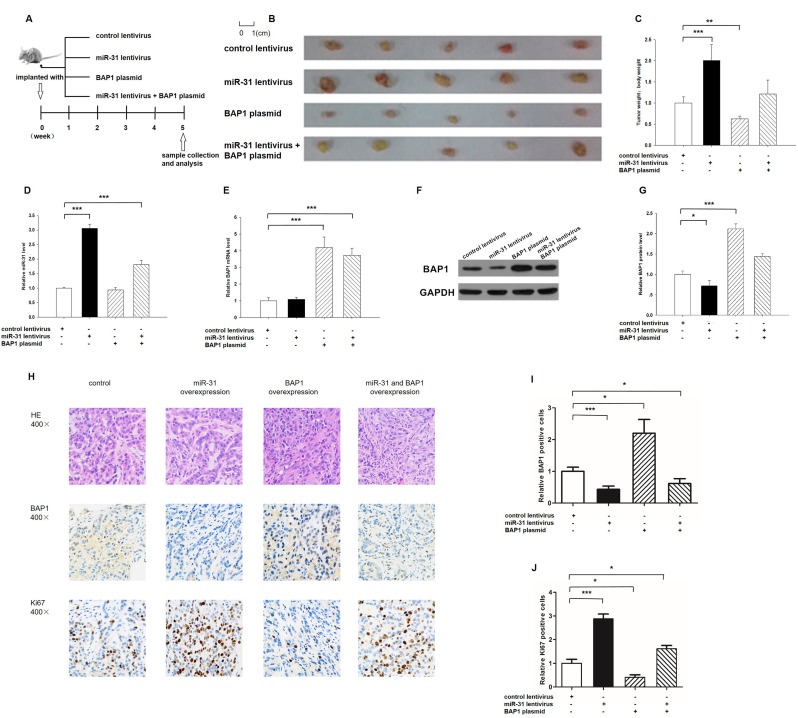
Effects of miR-31 and BAP1 on the growth of lung cancer cell xenografts in mice **A.** Flow chart of the experimental plan. A549 cells were infected with a control lentivirus or a lentivirus to overexpress miR-31, transfected with a BAP1 overexpression plasmid, or co-transfected with a lentivirus to overexpress miR-31 and a BAP1 overexpression plasmid. A549 cells (2 × 10^6^ cells per 0.1 mL) with different treatments were implanted subcutaneously into 6-week-old SCID mice (5 mice per group), and the tumor growth was evaluated on day 35 after cell implantation. **B.** Representative images of the tumors from the implanted mice. **C.** Quantitative analysis of the tumor weights. **D.** Quantitative RT-PCR analysis of miR-31 levels in the tumors from implanted mice. **E.** Quantitative RT-PCR analysis of BAP1 mRNA levels in the tumors from implanted mice. **F.** and **G.** Western blotting analysis of BAP1 protein levels in the tumors from implanted mice. **F.** representative image; **G.** quantitative analysis. **H.**-**J.** H&E-stained sections and immunohistochemical staining for Ki-67 and BAP1 in the tumors from implanted mice. **H.** representative image; **I.** and **J.** quantitative analysis. (* *p* < 0.05; ** *p* < 0.01; *** *p* < 0.005).

We evaluated the biological effects of miR-31 and BAP1 on the growth of lung cancer cells in a lung cancer xenograft mouse model. A 300-bp fragment containing the genomic miR-31 sequence was cloned into a lentiviral expression plasmid, and A549 cells were infected with the lentiviral plasmid to express miR-31. The efficient overexpression of miR-31 and the inhibition of BAP1 in A549 cells by lentiviral transfection is shown in Supplementary Figure 3A-3C. A549 cells were also transfected with a BAP1 overexpression plasmid. The efficient overexpression of BAP1 at protein levels is shown in Supplementary Figure 3B-3C. Subsequently, A549 cells (2 × 10^6^ cells per 0.1 mL) were infected with the miR-31 lentiviral expression plasmid, transfected with the BAP1 plasmid, or co-transfected with the miR-31 overexpression lentivirus plus BAP1 overexpression plasmid; then, the cells were implanted subcutaneously into 6-week-old SCID mice. After 35 days of xenograft growth *in vivo*, the mice were sacrificed and the weight of the tumors was measured (Figure 5A). A significant increase in the sizes and weights of the tumors was observed in the miR-31-overexpressing group compared to the control group, whereas the sizes and weight of the tumors in the group implanted with the BAP1-overexpression plasmid were dramatically decreased (Figure 5B and 5C). Additionally, BAP1 overexpression attenuated the promotive effect of miR-31 on tumor growth (Figure 5B and 5C), suggesting that miR-31 might promote tumor growth by silencing BAP1. Subsequently, total RNA and proteins were isolated from the tumors and analyzed. After 35 days of xenograft growth *in vivo*, tumors from the miR-31-overexpression group showed a significant increase in the expression of miR-31 compared to tumors from the control group (Figure 5D). Likewise, BAP1 mRNA levels were increased in the tumors from the BAP1-overexpressing group (Figure 5E). Tumors from the miR-31-overexpressing group displayed reduced BAP1 protein levels compared to tumors from the control group, whereas the tumors from the BAP1-overexpressing group showed elevated BAP1 protein levels (Figure 5F and 5G). Tumors with both miR-31 and BAP1 overexpression exhibited significantly higher levels of BAP1 compared to tumors with miR-31 overexpression (Figure 5F and 5G), suggesting that BAP1 overexpression could rescue the BAP1 suppression caused by miR-31. Furthermore, Hematoxylin and eosin (H&E) staining of xenograft tissues showed more cell mitosis in the group implanted with the miR-31 lentivirus compared with the control group, whereas confluent necrotic areas were observed in the xenografts from the BAP1-overexpressing group (Figure 5H). Xenografts with both miR-31 and BAP1 overexpression exhibited reduced cell mitosis compared to xenografts with miR-31 overexpression (Figure 5H), suggesting that BAP1 overexpression could attenuate the pro-proliferative effect of miR-31. Immunohistochemical staining also revealed the presence of lower levels of BAP1 in the tumors from mice implanted with miR-31-overexpressing cells, whereas the tumors from the BAP1-overexpressing mice showed increased BAP1 protein levels (Figure 5H and 5I). Finally, the proliferative activity of tumor cells was assessed by immunocytochemistry with the mouse monoclonal antibody Ki-67. The cell proliferation rate measured by the percentage of Ki-67-positive tumor cells was decreased in the group implanted with the BAP1 plasmid and increased in the group implanted with the miR-31 lentivirus (Figure 5H and 5J). Likewise, BAP1 overexpression attenuated the pro-proliferative effect caused by miR-31 overexpression (Figure 5H and 5J). These results were consistent with the findings of the *in vitro* assays, which firmly validated the role of miR-31 in promoting tumorigenesis through the suppression of BAP1.

## DISCUSSION

Lung cancer accounts for approximately 27% of all cancer deaths and 13% of all new cancers. Chemotherapy, radiotherapy and surgery are the three main lung cancer treatments, but the cure rate is very low. Recently, major advances in our comprehension of lung cancer biology have led to improved diagnostic and prognostic techniques and to the development of novel targeted therapies. However, the efficacy of the new treatments is limited by a combination of drug resistance and our limited understanding of tumor cell signaling pathways. Further understanding of the exact molecular mechanisms contributing to the pathogenesis of lung cancer is urgently needed for the development of novel therapeutics.

Recently, an important role for miRNAs in the genesis and progression of human cancers has emerged. Many researchers have reported the extensive alteration of miRNA expression patterns in the initial and developmental stages of human cancers. Therefore, miRNA profiling represents an invaluable tool for the classification of tumors that represent diagnostic challenges [21]. miRNAs may also serve as direct therapeutic targets for cancers. Overexpression of miRNAs can be silenced using antagomirs, and re-expression of miRNAs that are lost in cancers can be achieved by the overexpression of miRNA mimics. Some scientists have established the potential usefulness of miRNAs as therapeutic molecules against cancers, including the inhibition of cancer cell proliferation by miR-26a in a mouse model of hepatocellular carcinoma and the prevention of metastasis formation *via* the silencing of miR-10b. In this study, we showed that miR-31 was increased in lung cancer tissues. miR-31 promoted cell proliferation and suppressed cell apoptosis *in vitro* and accelerated tumor growth *in vivo*. Thus, it is quite possible that treatment with a miR-31 antagomir may be a promising strategy for lung cancers exhibiting an upregulation of miR-31 expression. In agreement with this hypothesis, miR-31 has also been shown to be increased and behave as an oncogenic miRNA in various human tumor types, including head and neck cancer [22], hepatocellular carcinoma and colorectal cancer [23]. Conversely, miR-31 may also act as a tumor-suppressive miRNA. For example, miR-31 is significantly decreased in breast cancer and could exert a tumor suppressor function in breast cancer by inhibiting RHOA [24]. Thus, whether miR-31 functions as an oncogene or tumor suppressor is dependent on the cell and tumor types; under different circumstances, miR-31 may exert different functions. Nevertheless, greater research emphasis is needed to characterize the feasibility of targeting miR-31 in lung cancer therapy and to develop simplified and cost-effective manipulation methods.

BAP1 is a deubiquitylase that takes part in multiple cellular pathways, including cell cycle, differentiation, cell death, gluconeogenesis, DNA damage repair and tumorigenesis [25]. BAP1 is located at the chromosome region 3p21.1 [26], which is a genomic region that is deleted in several human malignancies; accordingly, dysregulation of BAP1 has been reported in many types of cancers, such as uveal melanoma, meningioma and lung adenocarcinoma. For example, lung cancer cells with overexpressed BAP1 were reported to grow poorly and formed tumors that were approximately 10-15-fold smaller than those expressing BAP1 mutants when injected into mice [4]. BAP1 has a high somatic mutation rate in uveal melanoma and mesothelioma but very few mutations in lung cancer, suggesting that another regulation pathway may be involved in the dysregulation of BAP1 in lung cancer [27-31]. In this study, we showed that BAP1 was frequently decreased in lung cancer tissues. We also provided evidence that BAP1 suppressed cell proliferation and promoted cell apoptosis, suggesting that BAP1 tended to function as a tumor suppressor in lung cancer. Thus, BAP1 offers a particularly promising molecular target for lung cancer therapy. Unfortunately, technical limitations make it difficult to stably express BAP1 *in vivo*. In this study, we identified a novel regulatory network by employing miR-31 and BAP1 to fine-tune lung cancer cell proliferation and apoptosis and showed that binding BAP1 was one mechanism by which miR-31 exerted its oncogenic function. Considering that miR-31 is an upstream regulator of BAP1, it is possible to inhibit miR-31 to restore BAP1 expression *in vivo*.

Another issue that needs to be addressed is the levels of miR-31 in lung cancer tissues: are the 1.5-2 fold increases of miR-31 levels detected in Figure 1 sufficient to reduce the expression of BAP1 protein *in vivo*? In fact, it has been recently reported that transient transfection of miRNA mimics typically gives rise to supraphysiological miRNA levels in cells [32]. However, the supraphysiological levels of transfected miRNA do not represent the functional levels, because the majority of transfected miRNA is not associated with Argonaute as functionally active miRNAs, but is actually accumulated at non-functional locations such as lysosomes. Thus, although it was shown in Figure 3A that the levels of miR-31 were increased more than 500-fold in A549 cells after transfection, they cannot change so dramatically *in vivo*. To avoid the confusion, we have constructed lentiviral vector expressing miR-31 to produce functional intracellular miR-31 *via* the endogenous miRNA processing machinery. As shown in Supplementary Figure 3A, the expression levels of mature miR-31 were 2-fold higher than the basal levels when lung cancer cells were infected with miR-31-expressing lentivirus. Such fold change is biologically and physiological relevant, because the altered miR-31 also inhibited BAP1 expression and promoted the development of tumor (Figure 5F-5H), to the same degree as those obtained by using miR-31 mimics. Thus, the 1.5-2 fold increases of miR-31 levels in lung cancer tissues are sufficient to reduce the expression of BAP1 protein *in vivo*.

Taken together, this study not only revealed a critical role for miR-31 as an oncogenic miRNA in lung carcinogenesis but also explored the molecular mechanisms by which miR-31 contributed to lung cancer progression and identified BAP1 as a direct target gene of miR-31. Regulation of BAP1 by miR-31 might explain why the upregulation of miR-31 during lung carcinogenesis can promote cancer progression. Further research on miR-31 and BAP1 may reveal a new avenue for lung cancer treatment.

## MATERIALS AND METHODS

### Cells and human tissues

The human lung adenocarcinoma cell lines A549, H1975 and HCC827 were purchased from the Shanghai Institute of Cell Biology, Chinese Academy of Sciences (Shanghai, China). The cells were cultured in RPMI 1640 medium supplemented with 10% fetal bovine serum (FBS, Gibco, Carlsbad, CA, USA) in a 5% CO_2_, water-saturated atmosphere. The lung tumors and paired normal adjacent tissues were derived from patients undergoing a surgical procedure at the Nanjing Drum Tower Hospital, the Affiliated Hospital of Nanjing University Medical School (Nanjing, China). All of the patients provided written consent, and the Ethics Committee from Nanjing University approved all aspects of this study. Tissue fragments were immediately frozen in liquid nitrogen at the time of surgery and stored at −80°C. The clinical features of the patients are listed in Table 1.

### RNA isolation and quantitative RT-PCR

Total RNA was extracted from cultured cells and human tissues using the TRIzol Reagent (Sigma, St. Louis, MO, USA) according to the manufacturer's instructions. Assays to quantify miRNAs were performed using Taqman miRNA probes (Applied Biosystems, Foster City, CA, USA) according to the manufacturer's instructions. Briefly, 1 μg of total RNA was reverse-transcribed into cDNA using the AMV reverse transcriptase (TaKaRa, Dalian, China) and a stem-loop RT primer (Applied Biosystems). The reaction conditions were as follows: 16°C for 30 min, 42°C for 30 min, and 85°C for 5 min. Real-time PCR was performed using a TaqMan PCR kit on an Applied Biosystems 7300 Sequence Detection System (Applied Biosystems). The reactions were incubated in a 96-well optical plate at 95°C for 10 min, followed by 40 cycles of 95°C for 15 s and 60°C for 1 min. All of the reactions were run in triplicate. After the reaction, the cycle threshold (C_T_) data were determined using fixed threshold settings, and the mean C_T_ was determined from the triplicate PCRs. A comparative C_T_ method was used to compare each condition to the controls. The relative levels of miRNAs in the cells and tissues were normalized to U6. The amount of miRNA relative to the internal control U6 was calculated with the equation 2^−ΔΔCT^, in which ΔΔC_T_ = (C_T miR-31_ − C_T U6_)_target_ − (C_T miR-31_ − C_T U6_)_control_. To quantify BAP1 mRNA expression, 1 μg of total RNA was reverse-transcribed into cDNA using oligo dT and the AMV reverse transcriptase (TaKaRa, Dalian, China) with the following conditions: 16°C for 30 min, 42°C for 30 min, and 85°C for 5 min. Next, real-time PCR was performed with the RT product, SYBR Green Dye (Invitrogen) and specific primers for BAP1 and GAPDH. The sequences of the primers were as follows: BAP1 (sense): 5′-GACCCAGGCCTCTTCACC-3′; BAP1 (antisense): 5′-AGTCCTTCATGCGACTCAGG-3′; GAPDH (sense): 5′-CGAGCCACATCGCTCAGACA-3′; and GAPDH (antisense): 5′-GTGGTGAAGACGCCAGTGGA-3′. The reactions were incubated at 95°C for 5 min, followed by 40 cycles of 95°C for 30 s, 60°C for 30 s and 72°C for 1 min. After the reactions were completed, the C_T_ values were determined by setting a fixed threshold. The relative amount of BAP1 mRNA was normalized to GAPDH using a method similar to that described above.

### Protein extraction and western blotting

The cells and tissues were lysed in RIPA Lysis buffer (Beyotime, Shanghai, China) supplemented with a Protease and Phosphatase Inhibitor Cocktail (Thermo Scientific 78440) on ice for 30 min and then centrifuged for 10 min (12,000 x g, 4°C). The supernatant was collected, and the protein concentration was calculated with a Pierce BCA protein assay kit (Thermo Scientific, Rockford, IL, USA). The BAP1 protein levels were analyzed by Western blotting with a monoclonal anti-human BAP1 antibody (C-4, sc-28383). The protein levels were normalized by probing the same blots with a GAPDH antibody (FL-335, sc-25778). The anti-BAP1 and anti-GAPDH antibodies were purchased from Santa Cruz Biotechnology (CA, USA).

### Overexpression or knockdown of miR-31

miR-31 overexpression was achieved by transfecting lung cancer cells with a miRNA mimic (a synthetic RNA oligonucleotide duplex mimicking the miRNA precursor). Knockdown was achieved by transfecting a miRNA inhibitor (a chemically modified single-stranded antisense oligonucleotide designed to specifically sequester the mature miRNA). Synthetic pre-miR-31, anti-miR-31 and the scrambled negative control RNA (pre-miR-control and anti-miR-control) were purchased from GenePharma (Shanghai, China). A549, H1975 and HCC827 cells were seeded into 6-well plates or 60 mm dishes using RPMI 1640 media supplemented with 10% FBS. The cells were transfected with Lipofectamine 2000 (Invitrogen) using Opti-MEM Reduced Serum Medium(Gibco, Carlsbad, CA, USA) on the following day when the cells were approximately 60-70% confluent. Equal amounts of pre-miR-31 or pre-miR-control were used in each well. For the miRNA knockdown, equal amounts of anti-miR-31 or anti-miR-control were used. After 6 hours, the media was changed to RPMI 1640 supplemented with 2% FBS. The cells were harvested 24 h after the transfection and subjected to analysis by quantitative RT-PCR or Western blotting.

### Plasmid construction and siRNA interference assay

A mammalian expression plasmid encoding the full-length human BAP1 open reading frame without the miR-31-responsive 3′-UTR was purchased from Invitrogen. An empty plasmid served as the negative control. Three siRNA sequences binding different sites of the human BAP1 cDNA (si-BAP1) were designed and synthesized by GenePharma. A scrambled siRNA that did not bind the human BAP1 cDNA was synthesized as a negative control. The siRNA sequences were as follows: si-BAP1#1: 5′-CCGUGAUUGAUGAUGAUAUTT-3′ (sense); si-BAP1#2: 5′-CGGCCUUUCUAGACAAUCATT-3′ (sense); and si-BAP1#3: 5′-GGCUGAGAUUGCAAACUAUTT-3′ (sense). The BAP1 expression plasmid and BAP1 siRNA were transfected into A549 cells using Lipofectamine 2000 (Invitrogen) according to the manufacturer's instructions. Total RNA and protein were isolated 24 hours post-transfection. The BAP1 mRNA and protein expression levels were assessed by quantitative RT-PCR and Western blotting. The siRNA sequence with the best interfering effect (si-BAP1#3) was selected and used in this study.

### Luciferase reporter assay

To test the direct binding of miR-31 to the target gene BAP1, a luciferase reporter assay was performed as previously described [33]. A sequence containing the presumed miR-31 binding site was designed from the human BAP1 3′-UTR. The sequence was inserted into the p-MIR-reporter plasmid (Ambion). The insertion was confirmed to be correct by sequencing. To test the binding specificity, the sequences that were bound by the miR-31 seed sequence were mutated (from TCTTGCC to AGAACGG), and the mutant BAP1 3′-UTR was inserted into an equivalent luciferase reporter. For the luciferase reporter assays, A549 cells were cultured in 24-well plates, and each well was transfected with 0.4 μg of firefly luciferase reporter plasmid, 0.4 μg of a β-galactosidase (β-gal) expression plasmid (Ambion), and equal amounts (20 pmol) of pre-miR-31, anti-miR-31, or the scrambled negative control RNAs using Lipofectamine 2000 (Invitrogen). The β-gal plasmid was used as a transfection control. Twenty-four hours post-transfection, the cells were assayed using a luciferase assay kit (Promega, Madison, WI, USA).

### Cell proliferation assay

The proliferation of A549 cells was determined using the Cell Counting Kit-8 (Dojindo) according to the manufacturer's instructions. A549 cells were plated at a density of 5 × 10^3^ cells per well in 96-well plates and incubated overnight in RPMI 1640 medium supplemented with 10% FBS. The next day, the A549 cells were transfected with pre-miR-31, anti-miR-31, BAP1 siRNA or the overexpression plasmid, and the medium was changed to RPMI 1640 medium supplemented with 2% FBS. At 12, 24, 36 and 48 hours post-transfection, 10 μL CCK-8 liquid was added to the test well and incubated for 3 hours. The absorbance was measured at a wavelength of 450 nm.

### Cell apoptosis assay

Twenty-four hours after transfection with pre-miR-31, anti-miR-31, BAP1 siRNA or the overexpression plasmid, A549 cells were treated with RPMI 1640 medium without FBS for 24 hours to induce apoptosis. The cells were washed twice with cold PBS and resuspended in 1× binding buffer at a concentration of 1×10^6^ cells/mL according to the instructions of the FITC-Annexin V Apoptosis Detection Kit I (BD Biosciences). The cells (1×10^5^ cells) were transferred to a 5 mL culture tube, and FITC-Annexin V and propidium iodide (PI) were added. The cells were incubated for 15 min at room temperature in the dark and analyzed by flow cytometry (BD Biosciences) within 1 hour of staining.

### Establishment of tumor xenografts in mice

Six-week-old male SCID (severe combined immune deficiency) mice (nu/nu) were purchased from the Model Animal Research Center of Nanjing University (Nanjing, China) and maintained under specific pathogen-free conditions at Nanjing University. The mice were injected subcutaneously with A549 cells infected with a control lentivirus or a lentivirus to overexpress miR-31, transfected with a BAP1 overexpression plasmid, or co-transfected with a lentivirus to overexpress miR-31 and a BAP1 overexpression plasmid (2 × 10^6^ cells per mouse, 5 mice per group). The needle was inserted into the left side of the armpit, midway down, 5 mm deep, and at a 45° angle. The mice were sacrificed after 35 days. The mouse lung tumors were removed, and the weight of the tumors was measured. Parts of the tissues were used for protein and total RNA extraction, and the remainder were fixed in 4% paraformaldehyde for 24 h and then processed for Hematoxylin and eosin (H&E) staining and immunohistochemical staining for BAP1 and Ki-67.

### Statistical analysis

All of the Western blotting and cell apoptosis images are representative of at least three independent experiments. Quantitative RT-PCR, the luciferase reporter assay and the cell proliferation assay were performed in triplicate, and each experiment was repeated several times. The data shown are the mean ± SD or mean ± SE of at least three independent experiments. The differences were considered statistically significant at *p* < 0.05 using Student's t-test.

## SUPPLEMENTARY MATERIAL FIGURES


