# Supplementary material for: BAP1 suppresses lung cancer progression and is inhibited by miR-31
Source: Oncotarget. 2016 Feb 11;7(12):13742–53. doi: 10.18632/oncotarget.7328 (PMC4924675; doi:10.18632/oncotarget.7328)
Supplement: Supplementary file 1 [file oncotarget-07-13742-s001.pdf]

BAP1 suppresses lung cancer progression and is inhibited by miR-31

Supplementary Material

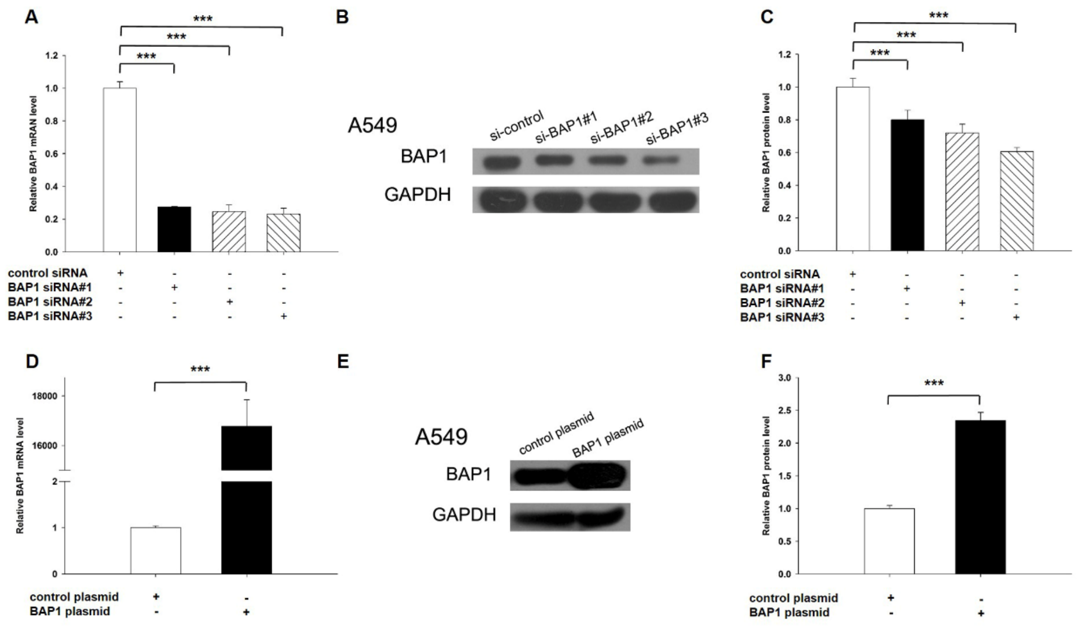

Supplementary Figure 1. Evaluation of the knockdown and overexpression efficiency of BAP1 in A549 cells. (A) Quantitative RT-PCR analysis of BAP1 mRNA levels in A549 cells transfected with three different BAP1 siRNAs or the scrambled negative control siRNA. **(B and C)** Western blotting analysis of the BAP1 protein level in A549 cells transfected with three different BAP1 siRNAs or the scrambled negative control siRNA. B: representative image; C: quantitative analysis. **(D)** Quantitative RT-PCR analysis of the BAP1 mRNA level in A549 cells transfected with the BAP1 overexpression plasmid or control plasmid. **(E and F)** Western blotting analysis of the BAP1 protein level in A549 cells transfected with the BAP1 overexpression plasmid or control plasmid. E: representative image; F: quantitative analysis. The results are presented as the mean ± S.D. of three independent experiments. (*** $p < 0.005$)

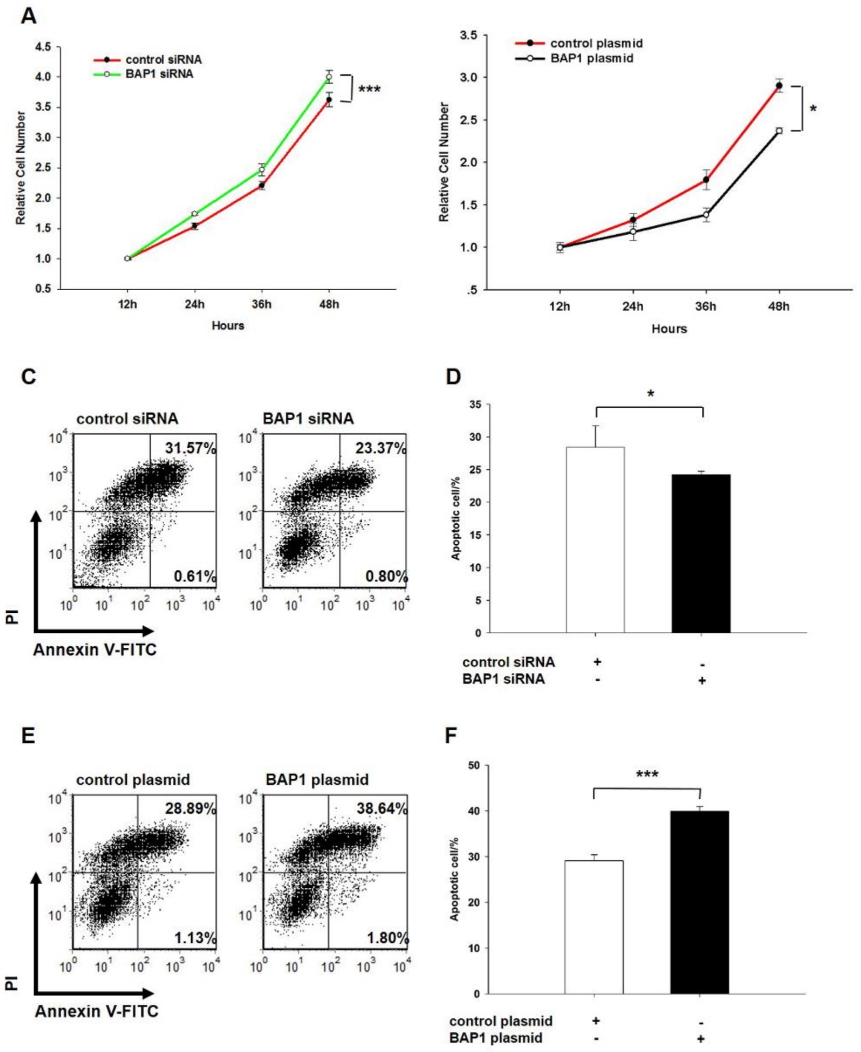

Supplementary Figure 2. Effect of BAP1 on the proliferation and apoptosis of lung cancer cells. (A) The cell proliferation assay was performed 12, 24, 36 and 48 hours after the transfection of A549 cells with the BAP1 siRNA or scrambled negative control siRNA. **(B)** The cell proliferation assay was performed 12, 24, 36 and 48 hours after the transfection of A549 cells with the BAP1 overexpression plasmid or control plasmid. **(C and D)** The apoptosis assay was performed 24 hours after the transfection of A549 cells with equal doses of the BAP1 siRNA or scrambled negative control siRNA. C: representative image; D: quantitative analysis. **(E and F)** The apoptosis assay was performed 24 hours after the transfection of A549 cells with equal doses of the BAP1 overexpression plasmid or control plasmid. E: representative image; F: quantitative analysis. The results of cell proliferation are presented as the mean ± S.E. of independent experience， and other results are presented as the mean ± S.D. of three independent experiments. (* $p < 0.05$; *** $p < 0.005$)

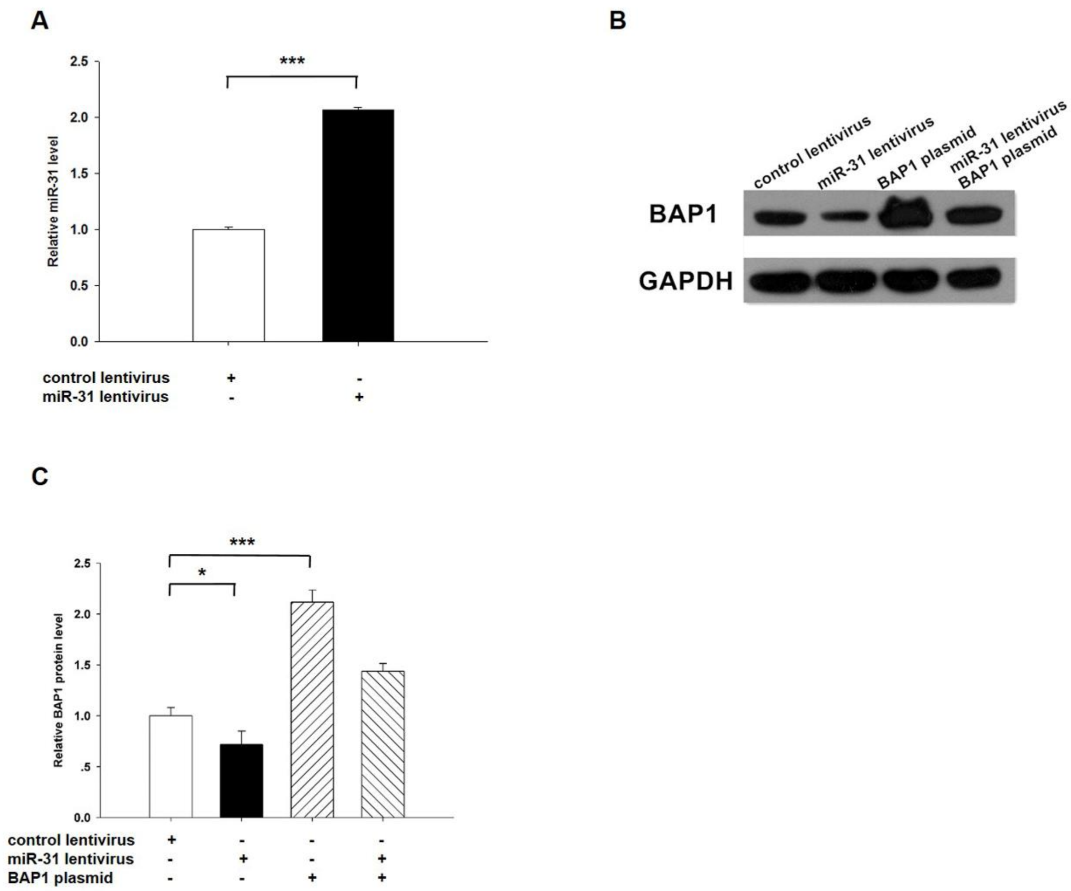

Supplementary Figure 3. Expression levels of BAP1 and miR-31 in A549 cells after transfection with a lentivirus to overexpress miR-31 or the BAP1 overexpression plasmid. (A) Quantitative RT-PCR analysis of miR-31 levels in A549 cells transfected with a control lentivirus or a lentivirus to overexpress miR-31. **(B and C)** Western blotting analysis of BAP1 protein levels in A549 cells transfected with a control lentivirus or a lentivirus to overexpress miR-31, transfected with a BAP1 overexpression plasmid, or co-transfected with a lentivirus to overexpress miR-31 and a BAP1 overexpression plasmid. B: representative image; C: quantitative analysis. (* $p < 0.05$; *** $p < 0.005$)
